# In water multicomponent synthesis of low-molecular-mass 4,7-dihydrotetrazolo[1,5-*a*]pyrimidines

**DOI:** 10.3762/bjoc.15.231

**Published:** 2019-10-08

**Authors:** Irina G Tkachenko, Sergey A Komykhov, Vladimir I Musatov, Svitlana V Shishkina, Viktoriya V Dyakonenko, Vladimir N Shvets, Mikhail V Diachkov, Valentyn A Chebanov, Sergey M Desenko

**Affiliations:** 1 State Scientific Institution "Institute for Single Crystals", National Academy of Sciences of Ukraine, Nauky Ave 60, Kharkiv 61072, Ukraine; 2 Karazin Kharkiv National University, Svobody Sq. 4, Kharkiv 61022, Ukraine; 3 Kharkiv Scientific Research Forensic Center, Ministry of Internal Affairs of Ukraine, 32 Kovtuna Str., Kharkiv 61036, Ukraine; 4 Zaporizhzhia State Medical University, Mayakovsky Ave 26, Zaporizhzhia 69035, Ukraine; 5 Bar-Ilan University Ramat Gan, 5290002, Israel

**Keywords:** 5-aminotetrazole, antioxidant activity, 1,3-dicarbonyl compounds, multicomponent synthesis, tetrazolo[1,5-*a*]pyrimidines

## Abstract

The three-component reaction of 5-aminotetrazole with aliphatic aldehydes (formaldehyde, acetaldehyde) and acetoacetic ester derivatives in water under microwave irradiation leads to the selective formation of 4,7-dihydrotetrazolo[1,5-*a*]pyrimidine derivatives. Under similar conditions using 4,4,4-trifluoroacetoacetic ester 5-hydroxy-4,5,6,7-tetrahydrotetrazolo[1,5-*a*]pyrimidines are obtained. The analogous reaction with acetylacetone requires scandium(III) triflate as catalyst. The antioxidant activity of selected compounds was assayed with 1,1-diphenyl-2-picrylhydrazyl.

## Introduction

Tetrazolo[1,5-*a*]pyrimidines and their partially hydrogenated derivatives are known for their interesting biological properties. They have been reported to have anticancer [1], antimicrobial [2–3] and antioxidant [3] activities and to act as inhibitors of hepatitis B virus [4]. The dihydro derivatives of tetrazolo[1,5-*a*]pyrimidines belong to a bit special kind of dihydropyrimidines due to the strong electron-withdrawing properties of the tetrazole ring, which makes them useful for studying various theoretical issues, e.g., tautomerism [5], intramolecular transformations [6], etc.

**Scheme 1:**
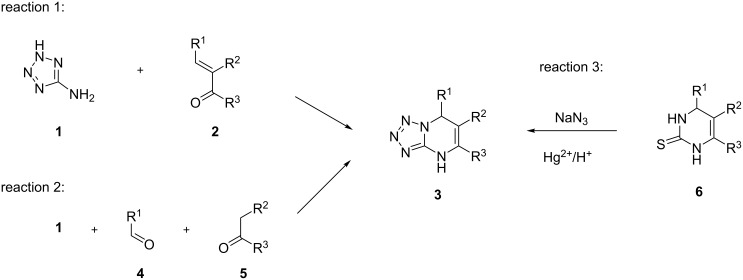
Three synthetic approaches to dihydrotetrazolo[1,5-*a*]pyrimidines.

There are several approaches to the synthesis of 4,7-dihydrotetrazolo[1,5-*a*]pyrimidines. Two of them make use of 5-aminotetrazole as a starting material and subsequent dihydropyrimidine ring formation. The first approach [6–7] represents a two-component cyclocondensation of 5-aminotetrazole (**1**) as binucleophilic component and bielectrophilic α,β-unsaturated carbonyl compounds **2** (Scheme 1, reaction 1). The second method [8–12] comprises the three-component reaction of amine **1** with the synthetic precursors of the unsaturated ketone **2** (Scheme 1, reaction 2), i.e., carbonyl compound **4** and a methylene-active compound **5**. These approaches are general and useful for the preparation of variety of dihydroazolopyrimidines which is easily accessible by variation of the binucleophilic component **1** (instead of **1**, 3-amino-1,2,4-triazole, 2-aminobenzimidazole, 3-aminopyrazoles, 4-amino-1,2,3-triazoles, etc. can be used [13]). However, a relatively low reactivity of amine **1** due to the electron deficiency of the tetrazole ring has been reported several times [5–6]. A third approach (Scheme 1, reaction 3) is completely different and consists of the tetrazole ring formation through cyclization of dihydropyrimidinethiones with sodium azide [14].

Generally, all three approaches allow for the preparation of a broad range of compounds **3** with a wide variety of substituents R^1^–R^3^. However, the vast majority of reported data [5–12,14–18] comprises aryl-substituted azolopyrimidines (with either R^1^ or R^3^ or both being aromatic substituents), and there are only a view examples of compounds **3** having no aryl substituent [19–20]. The reason for this relatively low synthetic availability might be due to their higher solubility (compared to aryl-substituted analogues) and difficulties associated with their isolation.

Further, to address green chemistry principles [21–24] for the synthesis of dihydrotetrazolopyrimidines one may concern carrying out these reactions under solvent-free conditions [15–17] or using water as a “green solvent” [18].

## Results and Discussion

### Synthesis

Due to the small number of reports available for dihydroazolopyrimidines with aliphatic substituents, we aimed by this work to synthesize a range of dihydrotetrazolo[1,5-*a*]pyrimidines containing no aromatic substituents. The second important reason for our interest in these compounds is based on the Lipinski rules for orally active drugs [25]. According to one of them, drug-like molecules should have molecular masses lower than 500 Da. Therefore, the minimization of molecular masses for the targeted tetrazolo[1,5-*a*]pyrimidines can be achieved by exclusion of large aryl substituents from their structures. Thus, we selected aliphatic aldehydes, i.e., formaldehyde and acetaldehyde, as the starting material and chose a multicomponent approach to minimize the number of reaction steps according to green chemistry principles as well. Based on our recent research [26–28], where water proved to be an effective solvent for the multicomponent synthesis of low-molecular-mass dihydroazolopyrimidines, we decided to use water also in this case.

**Scheme 2:**
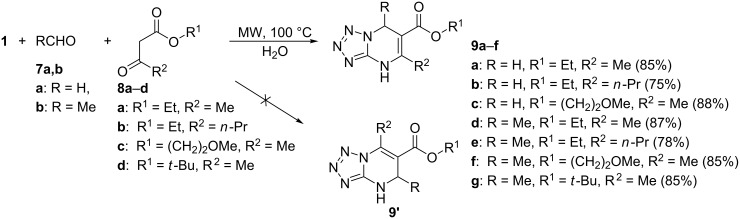
Three-component reaction of **1**, **7a**,**b** and **8a**–**d** in water.

The three-component reactions of 5-aminotetrazole (**1**) with aldehydes **7a**,**b** (paraformaldehyde, acetaldehyde) and a set of acetoacetic ester derivatives **8a**–**d** in water under microwave irradiation at 100 °C led to the formation of the corresponding 5,6,7-trisubstituted 4,7-dihydrotetrazolo[1,5-*a*]pyrimidines **9a**–**g** as single products. No formation of isomeric compounds **9’** was observed in any case (Scheme 2). Increasing the temperature (to 130 °C) and prolongation of the reaction time did not improve the yields of **9**, while decreasing the temperature to 80 °C resulted in a reduced yield.

**Figure 1:**
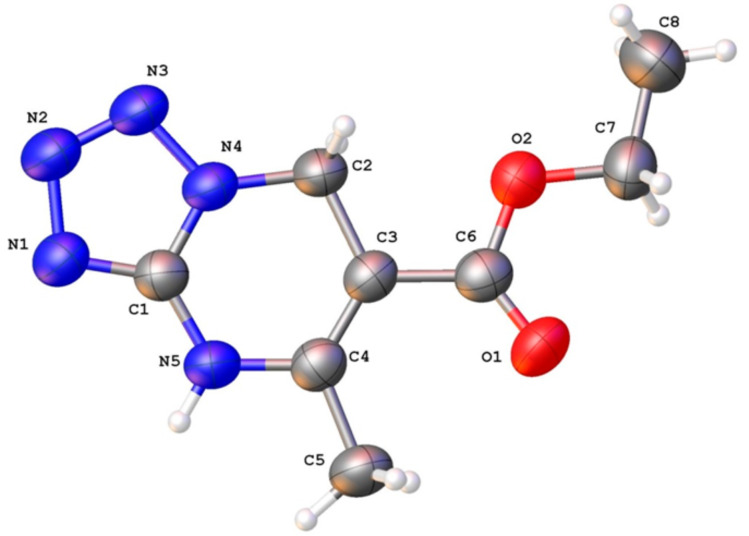
Molecular structure of **9a** according to X-ray data. Displacement ellipsoids are shown at the 50% probability level.

The structures of all products **9a**–**g** were proven by their spectral data. For example, the ^1^H NMR spectra of **9a**–**c** contained singlets for NH (10.84–10.90 ppm) and CH_2_ (5.06–5.08 ppm) protons that corresponded to the 4,7-dihydro structure of the tetrazolo[1,5-*a*]pyrimidines. Further, five basic signals were observed in the ^13^C NMR spectrum for the dihydrotetrazolopyrimidine bicycle: one signal was in the aliphatic area, three signals appeared at lower field, and one signal (C-6 of the bicycle) was observed between aliphatic and unsaturated carbons (92.5–92.7 ppm) which is typical for these compounds. Although the NMR data corresponded quite well to the proposed structure **9**, they did not allow the complete rejection of the isomeric structure **9'** for the reaction product. The final confirmation of structures **9a**–**g** was achieved by X-ray analysis of **9a** (Figure 1).

**Scheme 3:**
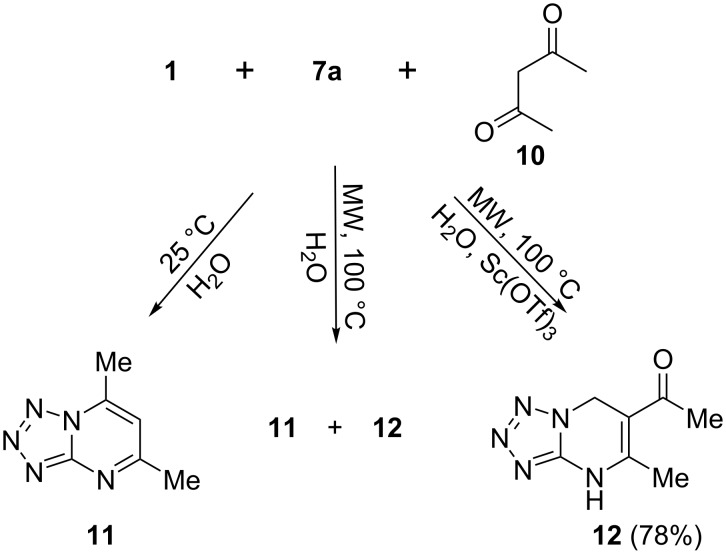
Three-component reaction of 5-aminotetrazole (**1**) with formaldehyde (**7a**) and acetylacetone (**10**).

A specific behavior of acetylacetone (**10**) as the 1,3-dicarbonyl compound was observed in the three-component reaction. Reacting it with amine **1** and aldehyde **7a** under conditions similar to those for acetoacetic esters (**8a**–**d**) led to formation of a mixture of compounds **11** and **12** (Scheme 3). Heteroaromatic tetrazolopyrimidine **11** was obtained as a single product from the three-component process performed at room temperature. Its structure based on NMR spectral data corresponded to a two-component condensation of amine **1** and acetylacetone (**10**). The selective formation of the target compound **12** was achieved in the presence of scandium(III) triflate as a catalyst.

**Scheme 4:**
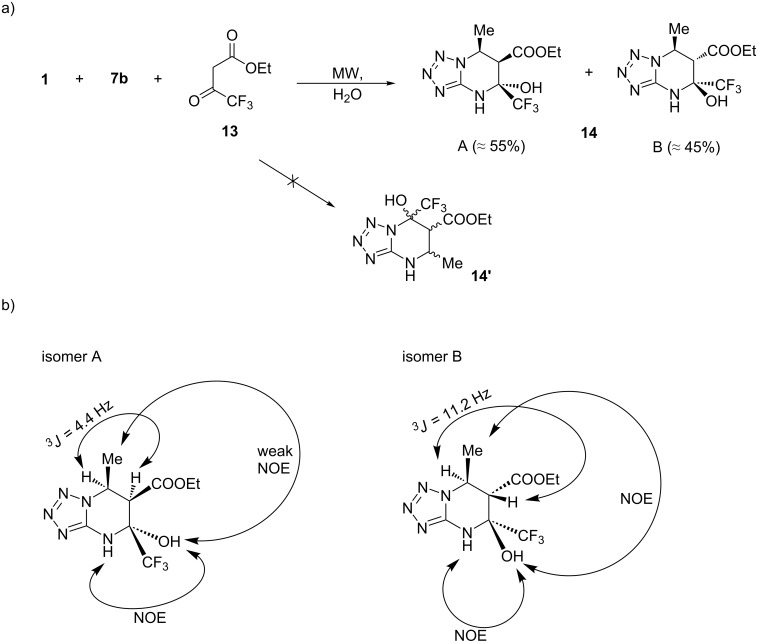
a) Three-component reaction of 5-aminotetrazole (**1**) with acetaldehyde (**7b**) and ethyl 4,4,4-trifluoroacetoacetate (**13**); b) structure investigation of **14** by NMR.

Another example of a 1,3-dicarbonyl compound that often exhibits unusual behavior in the three-component synthesis of azolopyrimidines [27,29] is ethyl 4,4,4-trifluoroacetoacetate (**13**). In these transformations, the last reaction step, a water elimination, sometimes does not occur, and the final product is the 5-hydroxy-containing tetrahydro derivative. In our experiments, the three-component reaction of amine **1** with aldehyde **7b** and compound **13** in water under microwave irradiation afforded tetrahydro derivative **14** as a mixture of two stereoisomers. Inspection of the mixture by ^1^H NMR revealed isomer **A** as the major component and isomer **B** as minor component in a ratio of ≈55:45. The ^1^H NMR spectrum of **14** contained a double set of signals for two methyl and one methylene group, two signals for methine protons and two singlets for NH and OH protons. The NOESY experiment allowed us to accomplish the final confirmation of the structure and to establish the relative stereochemistry of both stereoisomers. NOESY cross-peaks between signals of NH and OH protons revealed that **A** and **B** are in fact stereoisomers, rejecting the regioisomeric structure **14’**. The different ^3^*J* values between 6-H and 7-H for both isomers (4.4 Hz for **A** and 11.2 Hz for **B**) are indicative of different relative orientations of these protons and suggested that these protons in isomer **B** have *trans*-orientation, whereas those in isomer **A** have *cis*-orientation. Further, NOE between methyl and hydroxy groups for **B** isomer and a merely weak NOE for those groups in isomer **A** further allowed to propose a *cis*-orientation of these substituents for isomer **B** and the opposite one for **A** (Scheme 4).

### Antioxidant properties of **9**, **11**, **14**

Among the methods for the antioxidant activity (AOA) estimation, using 1,1-diphenyl-2-picrylhydrazyl (DPPH) is one of the most common and widespread [30–33]. The free radical scavenging activity of tetrazolo[1,5-*a*]pyrimidines **9d**,**f**, **11**, and **14** was measured spectrophotometrically as percentage of reducing the free-radical concentration in the presence of a test compound in methanol/dimethyl sulfoxide solutions. Ascorbic acid served as positive control. Among the tested compounds **9f** showed the best results at 10^−3^ mol/L concentration and compound **9d** exhibited the highest AOA at the other tested concentrations. The results of the radical scavenging experiments are collected in Table 1 and are promising for further detailed studies.

**Table 1:** Free radical scavenging activity of **9d**,**f**, **11**, and **14**.

Compound	AOA (%)		
	10^−3^ mol/L	10^−5^ mol/L	10^−7^ mol/L

**9d**	14.98	14.68	11.01
**9f**	19.10	2.64	0.06
**11**	15.30	9.35	6.80
**14**	13.89	3.47	0.00
ascorbic acid	92.17	60.15	64.43

## Conclusion

The three-component method which is based on the in-water reaction of 5-aminotetrazole with 1,3-dicarbonyl compounds and aliphatic aldehydes, like formaldehyde or acetaldehyde, under microwave activation, was applied for the preparation of low-molecular-mass 4,7-dihydrotetrazolo[1,5-*a*]pyrimidines. The use of acetoacetic ester derivatives as 1,3-dicarbonyl compounds in the reaction showed high selectivity under catalyst-free conditions, whereas in the case of acetylacetone the formation of a side product was observed. The desired product could be obtained with high selectivity by performing the reaction in the presence of scandium(III) triflate as a catalyst. 4,4,4-Trifluoromethylacetoacetic ester showed high reactivity in the current reaction forming the corresponding 5-hydroxy-5-trifluoromethyl-4,5,6,7-tetrahydrotetrazolo[1,5-*a*]pyrimidine as a mixture of isomers. Some of the prepared tetrazolopyrimidines showed free-radical scavenging activity towards DPPH.

## Experimental

**General.** The melting points were determined with a Gallenkamp melting point apparatus. The NMR spectra were recorded at 400 MHz with a Varian MR-400 spectrometer. The EIMS spectra were measured on a GC–MS Varian 1200L (ionizing voltage 70 eV, direct input of the sample) instrument. Elemental analysis was realized on a EuroVector EA-3000. Analytical samples of the compounds were obtained by crystallization from water and further drying at room temperature in air. Microwave experiments were performed using septum-sealed reaction vials in an Emrys Creator EXP from Biotage AB (Uppsala, Sweden) possessing a single-mode microwave cavity producing controlled irradiation at 2.45 GHz. Solvents and all reagents were commercially available and used without additional purification.

**Crystal data:** The crystal structure of compound **9a** was measured on an Xcalibur-3 diffractometer (graphite monochromated Mo K*_α_* radiation, CCD detector, *ω*-scanning). The structure was solved by the direct method using the SHELXTL package [34–35]. Full-matrix least-squares refinement against F^2^ in anisotropic approximation was used for non-hydrogen atoms. Positions of hydrogen atoms were determined from electron density difference maps and refined by ‘‘riding” model with U_iso_ = *n*U_eq_ of the carrier atom (*n* = 1.5 for methyl groups and *n* = 1.2 for other hydrogen atoms). Crystal data for C_8_H_11_N_5_O_2_ (**9a**) (*M* = 209.22 g/mol): triclinic, space group *P*−1, *a* = 4.2983(5) Å, *b* = 9.4739(8) Å, *c* = 13.1398(14) Å, α = 73.252(8)°, β = 88.290(9)°, *γ* = 79.628(8)°, *V* = 503.87(9) Å^3^, *Z* = 2, μ(Mo Kα) = 0.104 mm^−1^, *D*_calc_ = 1.379 g/cm^3^. Intensities were measured at 293 K within 2θ_max_ ≤ 54.992° (3905 reflections total, 2320 unique reflections, *R*_int_ = 0.031, R_sigma_ = 0.068). The final R_1_ = 0.062, wR_2_ = 0.136 for 2504 observed reflections with I ≥ 2σ(I) and R_1_ = 0.135, wR_2_ = 0.171 for all data, S = 1.024. Crystallographic data (excluding structure factors) for the structure of **9a** have been deposited with the Cambridge Crystallographic Data Centre as supplementary publication numbers CCDC 1942287. Copies of the data can be obtained, free of charge, on application to CCDC, 12 Union Road, Cambridge CB2 1EZ, UK, (fax: +44-(0)1223-336033 or by email: deposit@ccdc.cam.ac.uk).

**Free radical scavenging activity determination.** A solution of 0.1 mmol/L DPPH (2 mL) in methanol was added to 2 mL of a solution of the investigated substance (**9**, **11**, **14**) in dimethyl sulfoxide (DMSO) at different concentrations (10^−3^, 10^−5^, 10^−7^ mol/L). The control solution was prepared by mixing 2 mL of DMSO and 0.1 mmol/L DPPH solution (2 mL). The mixture was shaken vigorously and allowed to stand at room temperature for 30 min in the dark. The absorbance was measured at 517 nm using a spectrophotometer Specord-200. The percentage of DPPH scavenging was calculated as (AOA%) = [A_0_ − A_1_/A_0_] × 100, where A_0_ is the absorbance of the control solution, A_1_ is the absorbance of the test solution. Ascorbic acid dissolved in DMSO served as the reference compound.

**General procedure for the synthesis of 9a–g**. In a septum-sealed reaction vial, a solution of 5-aminotetrazole (**1**, 1.7 mmol), aldehyde **7** (paraformaldehyde **7a** or acetaldehyde **7b**, 0.15 g, ≈2 mmol) and acetoacetic ester derivative **8a–d** (1.77 mmol) in water (3.5 mL) was irradiated in a microwave reactor at 100 °C for 25–30 minutes. The crystalline product started to separate either during the course of reaction or just after cooling. The precipitate was filtered off, washed with water and air-dried.

**Ethyl 5-methyl-4,7-dihydrotetrazolo[1,5-*a*]pyrimidine-6-carboxylate (9a).** White solid, yield 85%; mp 200–202 °C; ^1^H NMR (400 MHz, DMSO-*d*_6_) δ 1.22 (t, ^3^*J*_HH_ = 6.8 Hz, 3H, CH_3_), 2.32 (s, 3Н, СН_3_), 4.11 (q, ^3^*J*_HH_ = 7.2 Hz, 2Н, CH_2_), 5.07 (s, 2Н, CН_2_), 10.87 (s, 1H, NH); ^13^C NMR (100 MHz, DMSO-*d*_6_) δ 14.2 (CH_3_), 18.1 (CH_3_), 44.1 (C-7), 59.8 (CH_2_), 92.7 (C-6), 146.7 (C-5), 148.9 (C-3a), 164.8 (CO); EIMS (70 eV, *m*/*z,* (%)): 209 (13) [M^+^], 181 (21), 109 (10); Anal. calcd for С_8_Н_11_N_5_O_2_ (209.09): C, 45.93; H, 5.30; N, 33.48%; found: C, 45.63; H, 5.07; N, 33.71%.

**Ethyl 5-propyl-4,7-dihydrotetrazolo[1,5-*a*]pyrimidine-6-carboxylate (9b).** White solid, yield 75%; mp 191–193 °C; ^1^H NMR (400 MHz, DMSO-*d*_6_) δ 0.92 (t, ^3^*J*_HH_ = 7.2 Hz, 3H, CH_3_), 1.22 (t, ^3^*J*_HH_ = 7.2 Hz, 3H, СН_3_), 1.57 (q, ^3^*J*_HH_ = 7.2 Hz, 2Н, CH_2_), 2.71 (t, ^3^*J*_HH_ = 7.6 Hz, 2Н, CН_2_), 4.12 (q, ^3^*J*_HH_ = 7.2 Hz, 2H, CH_2_), 5.08 (s, 2H, CH_2_), 10.84 (s, 1H, NH); ^13^C NMR (100 MHz, DMSO-*d*_6_) δ 13.6 (CH_3_), 14.1 (CH_3_), 21.6 (CH_2_), 32.7 (CH_2_), 44.2 (C-7), 59.8 (CH_2_), 92.5 (C-6), 149.0 (C-5), 150.6 (C-3a), 164.6 (CO); EIMS (70 eV, *m/z* (%)): 237 (2) [M^+^], 137 (13), 111 (10), 109 (19); Anal. calcd for С_10_Н_15_N_5_O_2_ (237.12): C, 50.62; H, 6.37; N, 29.52%; found: C, 50.42; H, 6.54; N, 29.38%.

**2-Methoxyethyl 5-methyl-4,7-dihydrotetrazolo[1,5-*a*]pyrimidine-6-carboxylate (9c)** White solid, yield 88%; mp 107–109 °C; ^1^H NMR (400 MHz, DMSO-*d*_6_) δ 2.33 (s, 3Н, СН_3_), 3.27 (s, 3Н, CH_3_), 3.56 (t, ^3^*J*_HH_ = 4.8 Hz, 2Н, CН_2_), 4.20 (t, ^3^*J*_HH_ = 4.8 Hz, 2H, CH_2_), 5.07 (s, 2H, CH_2_), 10.89 (s, 1H, NH); ^13^C NMR (100 MHz, DMSO-*d*_6_) δ 18.1 (CH_3_), 44.1 (C-7), 58.1 (CH_3_) , 62.8 (CH_2_), 69.8 (CH_2_), 92.5 (C-6), 147.0 (C-5), 148.9 (C-3a), 164.8 (CO); EIMS (70 eV, *m/z* (%)): 239 (12) [M^+^], 238 (100); Anal. calcd for С_9_Н_13_N_5_O_3_ (239.23): C, 45.18; H, 5.48; N, 29.27%; found: C, 45.27; H, 5.65; N, 29.09%.

**Ethyl 5,7-dimethyl-4,7-dihydrotetrazolo[1,5-*a*]pyrimidine-6-carboxylate (9d).** White solid, yield 87%; mp 173–175 °C; ^1^H NMR (400 MHz, DMSO-*d*_6_) δ 1.24 (t, ^3^*J*_HH_ = 7.2 Hz, 3H, CH_3_), 1.47 (d, ^3^*J*_HH_ = 6.4 Hz, 3Н, СН_3_), 2.32 (s, 3Н, CH_3_), 4.15 (m, 2Н, CН_2_), 5.66 (q, ^3^*J*_HH_ = 6.0 Hz, 1Н, Н); 10.96 (s, 1H, NH); ^13^C NMR (100 MHz, DMSO-*d*_6_) δ 14.1 (CH_3_), 18.4 (CH_3_), 23.0 (CH_3_), 51.5 (C-7), 59.8 (CH_2_), 98.4 (C-6), 146.3 (C-5), 148.6 (C-3a), 164.8 (CO); EIMS (70 eV, *m/z* (%)): 223 (10) [M^+^], 222 (100); Anal. calcd for С_9_Н_13_N_5_O_2_ (223.11): C, 48.42; H, 5.87; N, 31.37%; found: C, 48.60; H, 5.65; N, 31.52%.

**Ethyl 7-methyl-5-propyl-4,7-dihydrotetrazolo[1,5-*a*]pyrimidine-6-carboxylate (9e).** Colorless solid, yield 78%; mp 137–139 °C; ^1^H NMR (400 MHz, DMSO-*d*_6_) δ 0.91 (t, ^3^*J*_HH_ = 7.2 Hz, 3H, CH_3_), 1.24 (t, ^3^*J*_HH_ = 7.2 Hz, 3Н, СН_3_), 1.46 (d, ^3^*J*_HH_ = 6.4 Hz, 3H, CH_3_), 1.57 (m, 2Н, CH_2_), 2.69 (m, 2Н, CН_2_), 4.15 (m, 2H, CH_2_), 5.67 (q, ^3^*J*_HH_ = 6.0 Hz, 1H, CH), 10.96 (s, 1H, NH); ^13^C NMR (100 MHz, DMSO-*d*_6_) δ 13.6 (CH_3_), 14.1 (CH_3_), 21.6 (CH_3_), 23.1 (CH_2_), 32.9 (CH_2_), 51.5 (C-7), 59.9 (CH_2_), 98.3 (C-6), 148.7 (C-5), 150.2 (C-3a), 164.6 (CO); EIMS (70 eV, *m/z* (%)): 251 (18) [ M^+^], 250 (100); Anal. calcd for С_11_Н_17_N_5_O_2_ (251.14): C, 52.58; H, 6.82; N, 27.87%; found: C, 52.69; H, 6.74; N, 27.96%.

**2-Methoxyethyl 5,7-dimethyl-4,7-dihydrotetrazolo[1,5-*a*]pyrimidine-6-carboxylate (9f)** Colorless solid, yield 85%; mp 138–140 °C; ^1^H NMR (400 MHz, DMSO-*d*_6_) δ 1.48 (d, ^3^*J*_HH_ = 6.4 Hz, 3Н, СН_3_), 2.32 (s, 3Н, CH_3_), 3.27 (s, 3Н, CН_3_), 3.57 (t, ^3^*J*_HH_ = 4.8 Hz, 2H, CH_2_), 4.15 (m, 1H, CH_2_), 4.29 (m, 1H, CH_2_), 5.65 (q, ^3^*J*_HH_ = 6.0 Hz, 1H, CH), 10.99 (s, 1H, NH); ^13^C NMR (100 MHz, DMSO-*d*_6_) δ 18.5 (CH_3_), 23.0 (CH_3_), 51.6 (C-7), 58.1 (CH_3_), 62.8 (CH_2_), 69.9 (CH_2_), 98.2 (C-6), 146.8 (C-5), 148.6 (C-3a), 164.8 (CO); EIMS (70 eV, *m/z* (%)): 253 (13) [M^+^], 252 (100); Anal. calcd for С_10_Н_15_N_5_O_3_ (253.26): C, 47.42; H, 5.97; N, 27.65%; found: C, 47.53; H, 5.88; N, 27.76%.

***tert*-Butyl 5,7-dimethyl-4,7-dihydrotetrazolo[1,5-*a*]pyrimidine-6-carboxylate (9g)** Colorless solid, yield 85%; mp 174–175 °C; ^1^H NMR (400 MHz, DMSO-*d*_6_) δ 1.43 (s, 9Н, 3СН_3_), 1.44 (d, ^3^*J*_HH_ = 1.6 Hz, 3Н, CH_3_), 2.26 (s, 3Н, CН_3_), 5.57 (q, ^3^*J*_HH_ = 6.0 Hz, 1H, CH), 10.82 (s, 1H, NH); ^13^C NMR (100 MHz, DMSO-*d*_6_) δ 18.4 (CH_3_), 22.9 (CH_3_), 27.9(3CH_3_-*t*-Bu), 51.6 (C-7), 80.2 (C-*t*-Bu), 99.6 (C-6), 145.3 (C-5), 148.6 (C-3a), 164.2 (CO); EIMS (70 eV, *m/z* (%)): 251 (19) [M^+^], 250 (100); Anal. calcd for С_11_Н_17_N_5_O_2_ (251.28): C, 52.58; H, 6.82; N, 27.87%; found: C, 52.69; H, 6.92; N, 27.78%.

**1-(5-Methyl-4,7-dihydrotetrazolo[1,5-*a*]pyrimidin-6-yl)ethanone (12).** In a vial, a solution of 5-aminotetrazole (**1**, 1.2 mmol, 0.1 g), paraformaldehyde (**7a**, 1.26 mmol, 0.038 g), acetylacetone (**10**, 1.2 mmol, 0.122 ml) and 0.012 g scandium(III) triflate (Sc(OTf)_3_·*n*H_2_O) in water (3.2 mL) was irradiated in a microwave reactor at 100 °C for 20 min. The crystalline product started to separate after cooling to 5 °C. The resulting precipitate was filtered off, washed with water and air-dried. Colorless solid, yield 78%; mp 165–167 °C; ^1^H NMR (400 MHz, DMSO-*d*_6_) δ 2.25 (s, 3H, СН_3_), 2.32 (s, 3Н, СН_3_), 5.2 (s, 2Н, CН_2_), 10.84 (s, 1H, NH); ^13^C NMR (100 MHz, DMSO-*d*_6_) δ 19.2 (CH_3_), 30.3 (CH_3_), 44.6 (C-7), 102.9 (C-6), 145.5 (C-5), 148.8 (C-3a), 194.6 (CO); EIMS (70 eV, *m/z* (%)): 179 (15) [ M^+^], 178 (100), 122 (32); Anal. calcd for С_7_Н_9_N_5_O (179.08): C, 46.92; H, 5.06; N, 39.09%; found: C, 47.20; H, 5.35; N, 39.36%.

**Ethyl (5*RS*,6*RS*,7*SR*)-5-hydroxy-7-methyl-5-(trifluoromethyl)-4,5,6,7-tetrahydrotetrazolo[1,5-*a*]pyrimidine-6-carboxylate and ethyl (5*SR*,6*SR*,7*SR*)-5-hydroxy-7-methyl-5-(trifluoromethyl)-4,5,6,7-tetrahydrotetrazolo[1,5-*a*]pyrimidine-6-carboxylate (14).** In a vial, a solution of 5-aminotetrazole (**1**, 1.7 mmol), acetaldehyde (**7b**, 0.15 g, ≈2 mmol) and ethyl 4,4,4-trifluoroacetoacetate (**13**, 1.77 mmol) in water (3.5 mL) was irradiated in a microwave reactor at 100 °C for 25–30 min. The crystalline product started to separate either during the course of reaction or just after cooling. The precipitate was filtered, washed with water and air-dried. White solid, yield 90%; mp 139–140 °C; ^1^H NMR (400 MHz, DMSO-*d*_6_) isomer A: δ 1.08 (t, ^3^*J*_HH_ = 7.2 Hz, 3H, CH_3_), 1.59 (d, ^3^*J*_HH_ = 6.4 Hz, 3Н, CH_3_), 3.28 (d, ^3^*J*_HH_ = 4.4 Hz, 1Н, CН), 4.02 (q, ^3^*J*_HH_ = 7.2 Hz, 2H, CH_2_), 4.76 (m, 1H, CH), 7.77 (s, 1H, OH), 9.49 (s, 1H, NH); isomer B: 1.19 (t, ^3^*J*_HH_ = 7.2 Hz, 3H, CH_3_), 1.59 (d, ^3^*J*_HH_ = 6.4 Hz, 3Н, CH_3_, 3.17 (d, ^3^*J*_HH_ = 11,2 Hz, 1Н, CН), 4.17 (q, ^3^*J*_HH_ = 7.2 Hz, 2H, CH_2_), 4,63 (m, 1H, CH), 7,89 (s, 1H, OH), 9.34 (s, 1H, NH); ^13^C NMR (100 MHz, DMSO-*d*_6_) isomer A: δ 13.7 (CH_3_), 16.2 (7-CH_3_) , 46.9 (C-7), 49.3 (C-6), 61.4 (CH_2_) , 80.8 (C-5, q, ^2^*J*_CC_= 31.0 Hz), 123.0 (q, ^1^*J*_CF_ = 286.0 Hz), 151.6 (C-3*a*), 167.0 (CO); isomer B: δ 13.7 (CH_3_), 14.3 (7-CH_3_), 48.2 (C-7), 49.0 (C-6), 61.0 (CH_2_), 80.8 (C-5, q, ^2^*J*_CC_= 32.0 Hz), 123.0 (q, ^1^*J*_CF_ = 286.0 Hz), 152.3 (C-3*a*), 166.3 (CO); EIMS (70 eV, *m/z* (%)): 295 (15) [M^+^], 294 (100), 277 (12), 276 (60), 180 (25); Anal. calcd for С_9_Н_12_F_3_N_5_O_3_ (295.23): C, 36.62; H, 4.10; F, 19.31; N, 23.72%; found: C, 36.53; H, 4.21; F, 19.20; N, 23.84%.

## Supporting Information

File 1 Copies of ^1^H and ^13^C NMR spectra for **9a**–**g**, **12**, and **14**.

File 2 Crystal data of **9a**.
