# Supplementary material for: In water multicomponent synthesis of low-molecular-mass 4,7-dihydrotetrazolo[1,5-a]pyrimidines
Source: Beilstein J Org Chem. 2019 Oct 8;15:2390–7. doi: 10.3762/bjoc.15.231 (PMC6808202; doi:10.3762/bjoc.15.231)
Supplement: File 1 — Copies of 1H and 13C NMR spectra for 9a–g, 12, and 14. [file Beilstein_J_Org_Chem-15-2390-s001.pdf]

BEILSTEIN JOURNAL OF ORGANIC CHEMISTRY

Supporting Information

for

In water multicomponent synthesis of low-molecular-mass 4,7-dihydrotetrazolo[1,5-*a*]pyrimidines

Irina G. Tkachenko, Sergey A. Komykhov, Vladimir I. Musatov, Svitlana V. Shishkina, Viktoriya V. Dyakonenko, Vladimir N. Shvets, Mikhail V. Diachkov, Valentyn A. Chebanov and Sergey M. Desenko

Beilstein J. Org. Chem. **2019,** *15,* 2390–2397. doi:10.3762/bjoc.15.231

Copies of ^{1}H and ^{13}C NMR spectra for 9a–g, 12, and 14

License and Terms: This is a supporting information file under the terms of the Creative Commons Attribution License (http://creativecommons.org/licenses/by/4.0). Please note that the reuse, redistribution and reproduction in particular requires that the authors and source are credited.
The license is subject to the *Beilstein Journal of Organic Chemistry* terms and conditions: (https://www.beilstein-journals.org/bjoc)

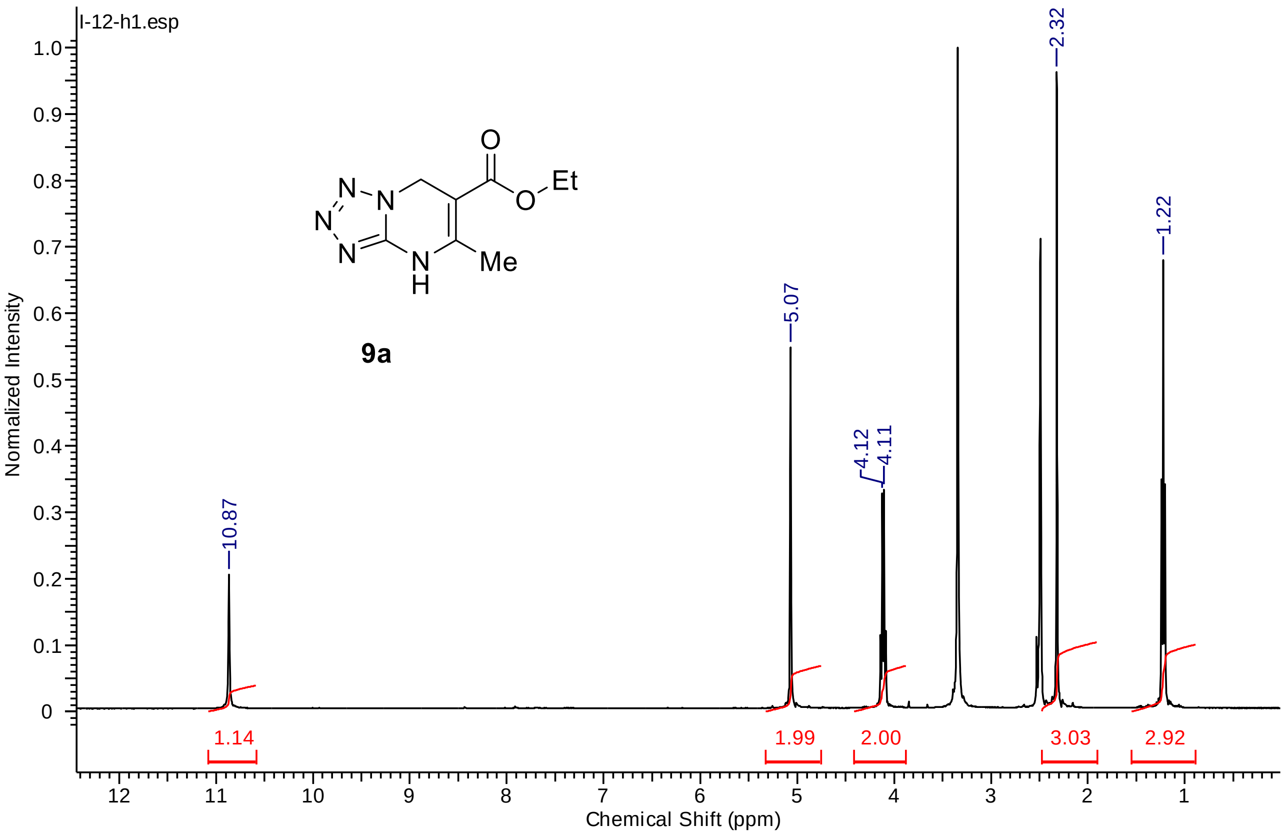
l-12-h1.esp
9a
O
Et
N
Me
H
10.87
5.07
4.12
4.11
2.32
1.22
1.14
1.99
2.00
3.03
2.92
Normalized Intensity
Chemical Shift (ppm)

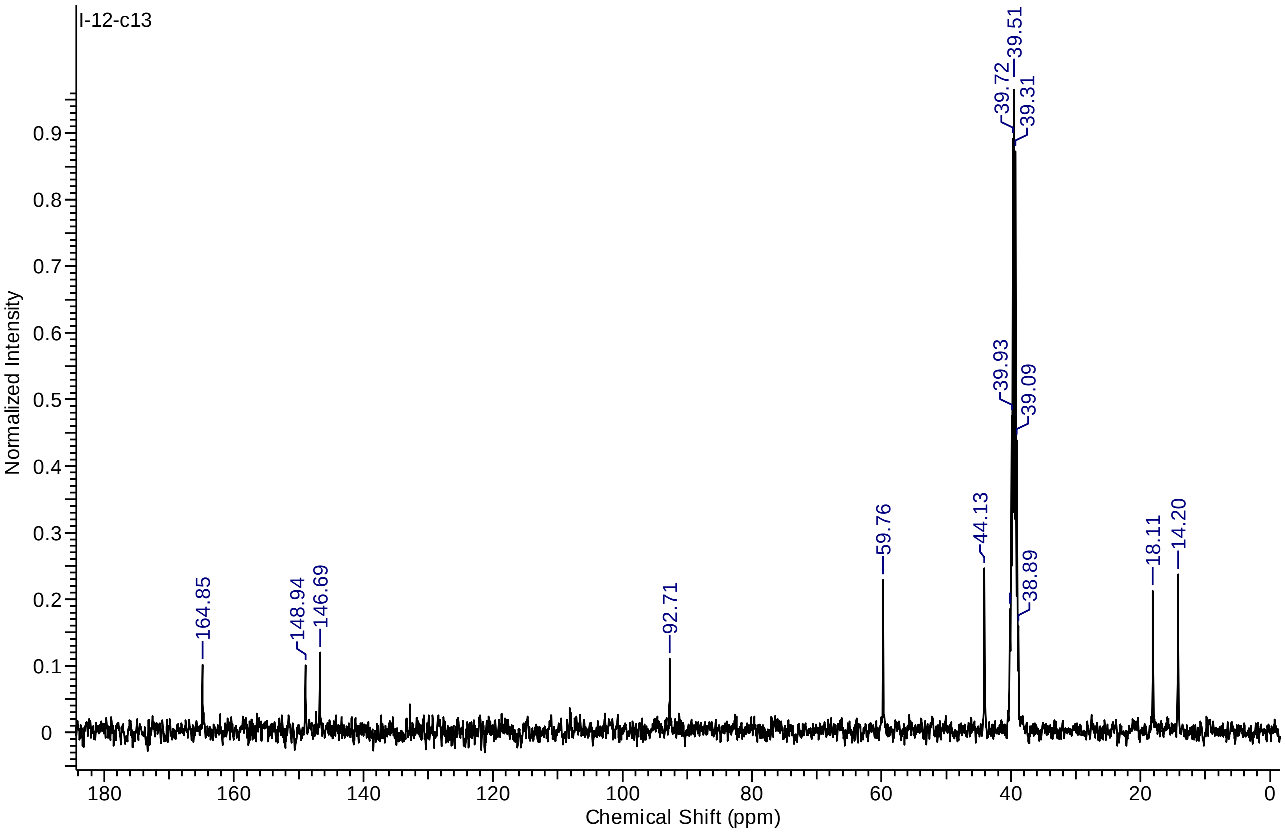
l-12-c13
164.85
148.94
146.69
92.71
59.76
44.13
39.93
39.72
39.51
39.31
39.09
38.89
18.11
14.20
Normalized Intensity
Chemical Shift (ppm)

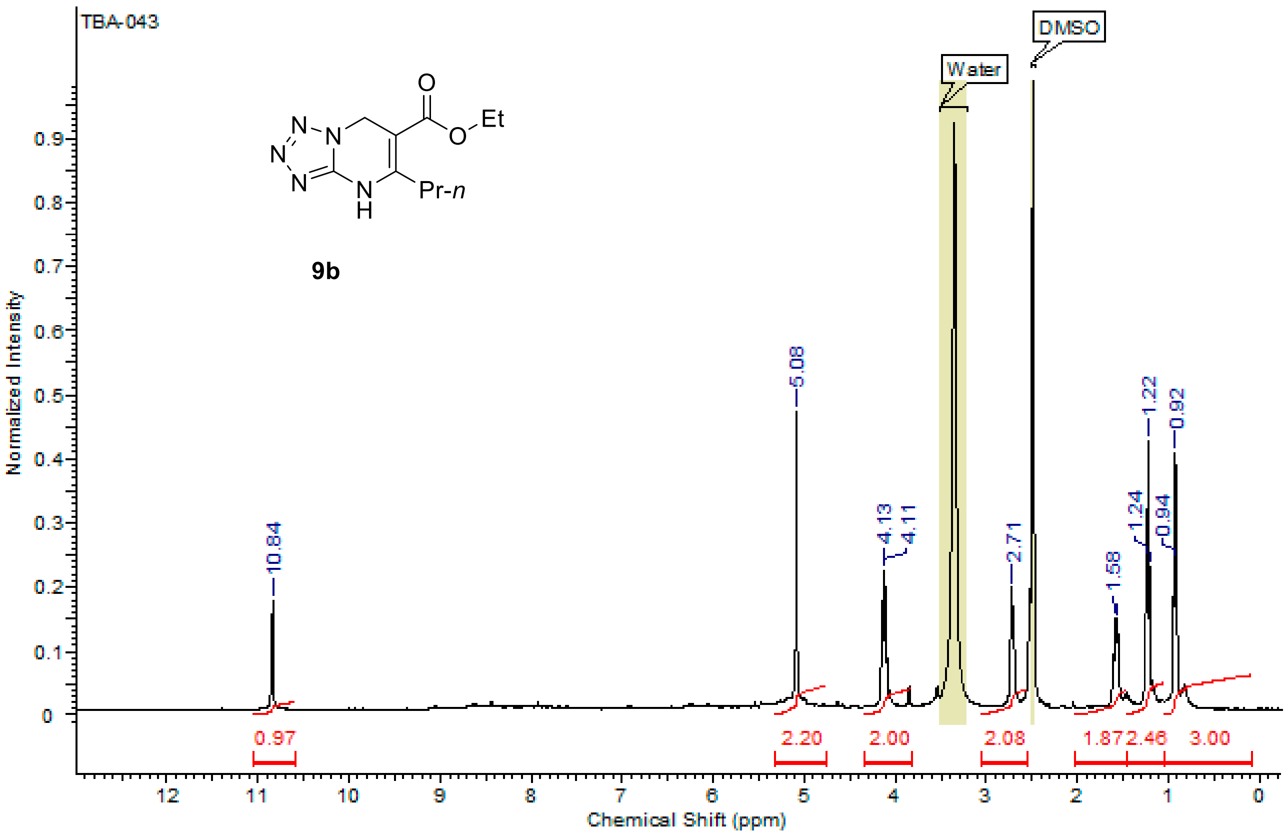
TBA-043
Water
DMSO
9b
O
Et
N
Pr-n
H
10.84
5.08
4.13
4.11
2.71
1.58
1.24
1.22
0.94
0.92
0.97
2.20
2.00
2.08
1.87
2.46
3.00
Normalized Intensity
Chemical Shift (ppm)

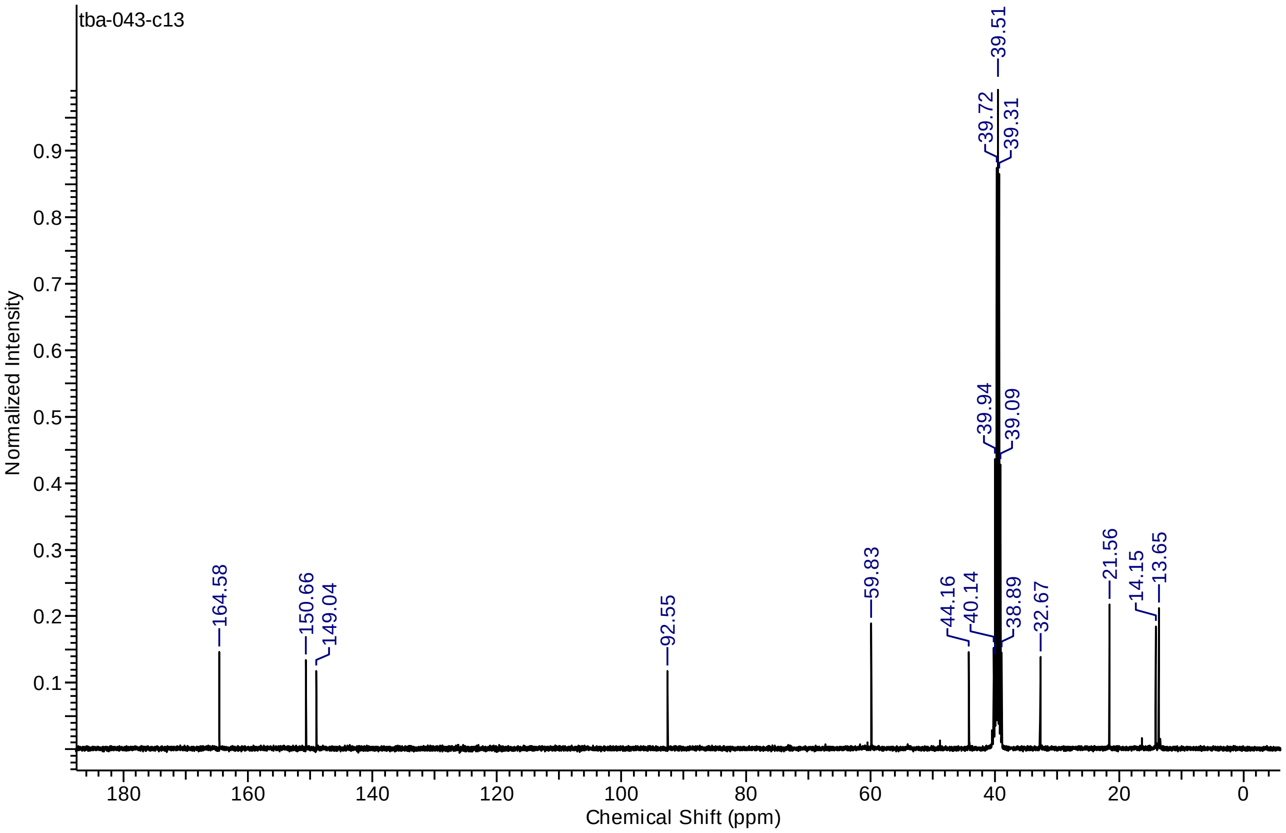
tba-043-c13
164.58
150.66
149.04
92.55
59.83
44.16
40.14
39.94
39.72
39.51
39.31
39.09
38.89
32.67
21.56
14.15
13.65
Normalized Intensity
Chemical Shift (ppm)

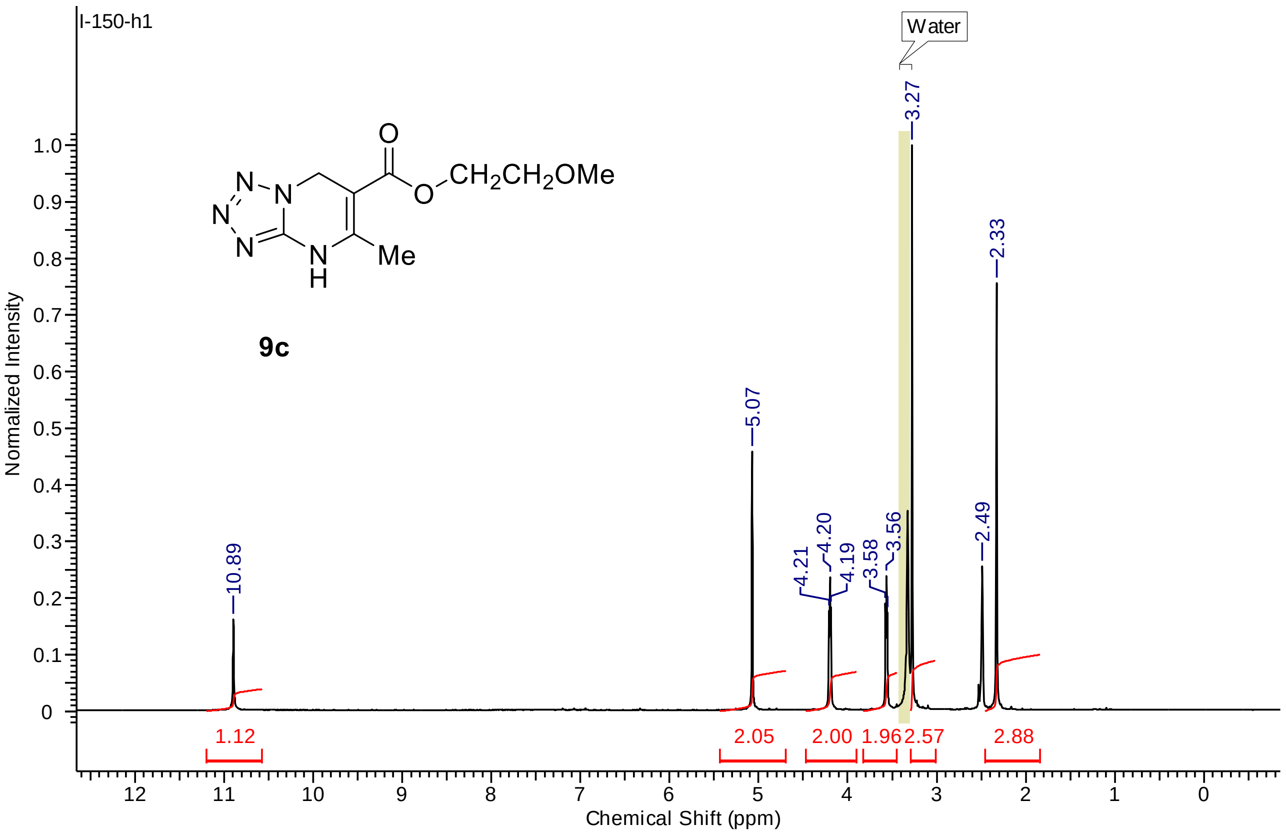
I-150-h1
Water
9c
CH2CH2OMe
Me
10.89
5.07
4.21
4.20
4.19
3.58
3.56
3.27
2.49
2.33
1.12
2.05
2.00
1.96
2.57
2.88
Normalized Intensity
Chemical Shift (ppm)

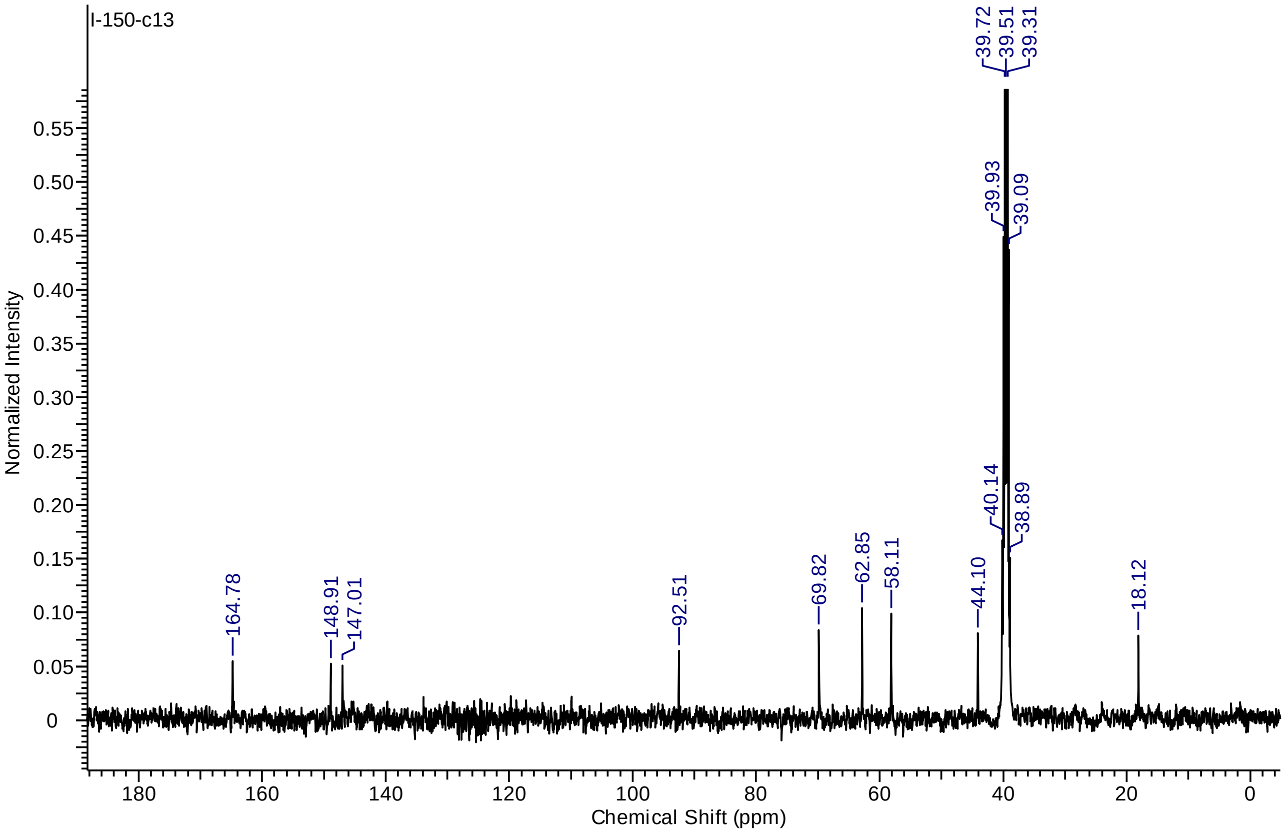
I-150-c13
164.78
148.91
147.01
92.51
69.82
62.85
58.11
44.10
40.14
39.93
39.72
39.51
39.31
39.09
38.89
18.12
Normalized Intensity
Chemical Shift (ppm)

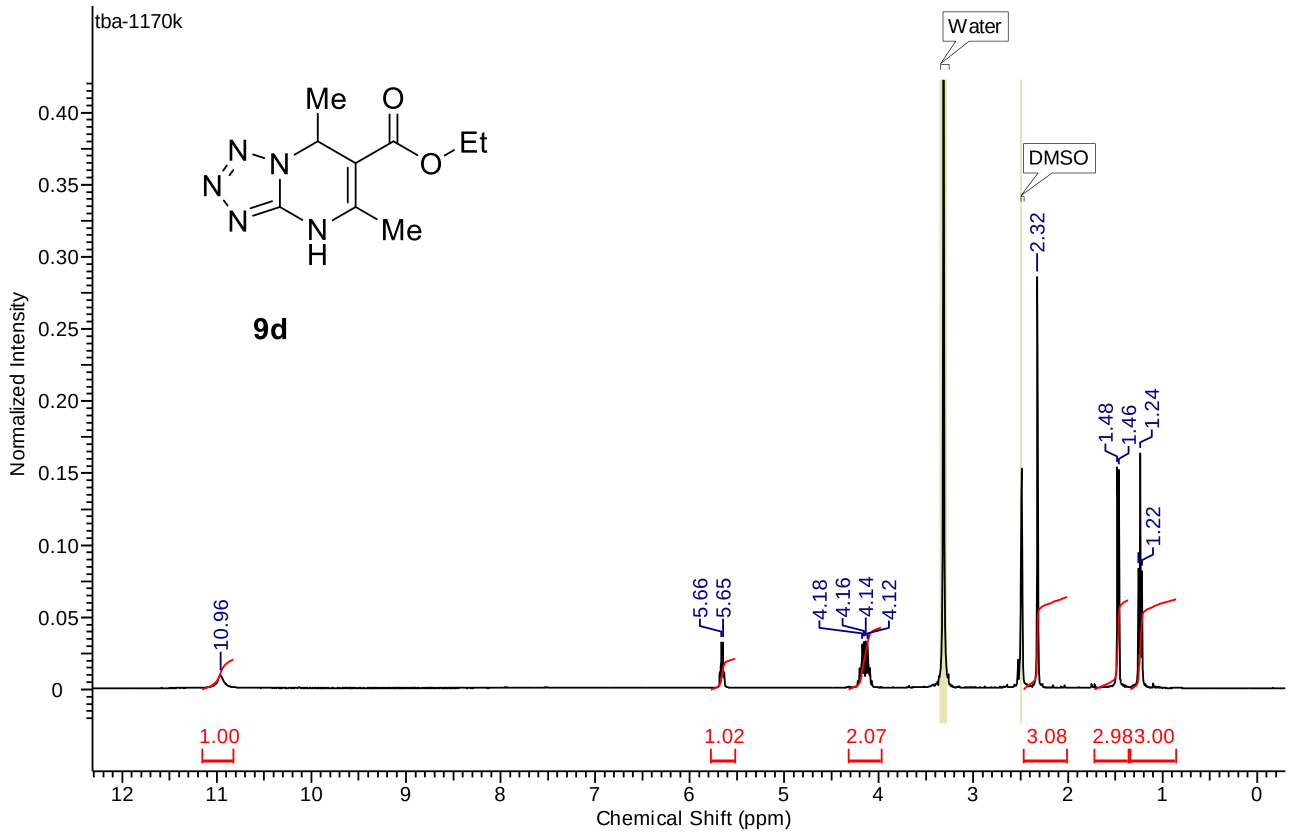
tba-1170k
Water
DMSO
Me
O
N
N
N
N
O
Et
N
H
Me
9d
Normalized Intensity
10.96
5.66
5.65
4.18
4.16
4.14
4.12
2.32
1.48
1.46
1.24
1.22
1.00
1.02
2.07
3.08
2.98
3.00
Chemical Shift (ppm)

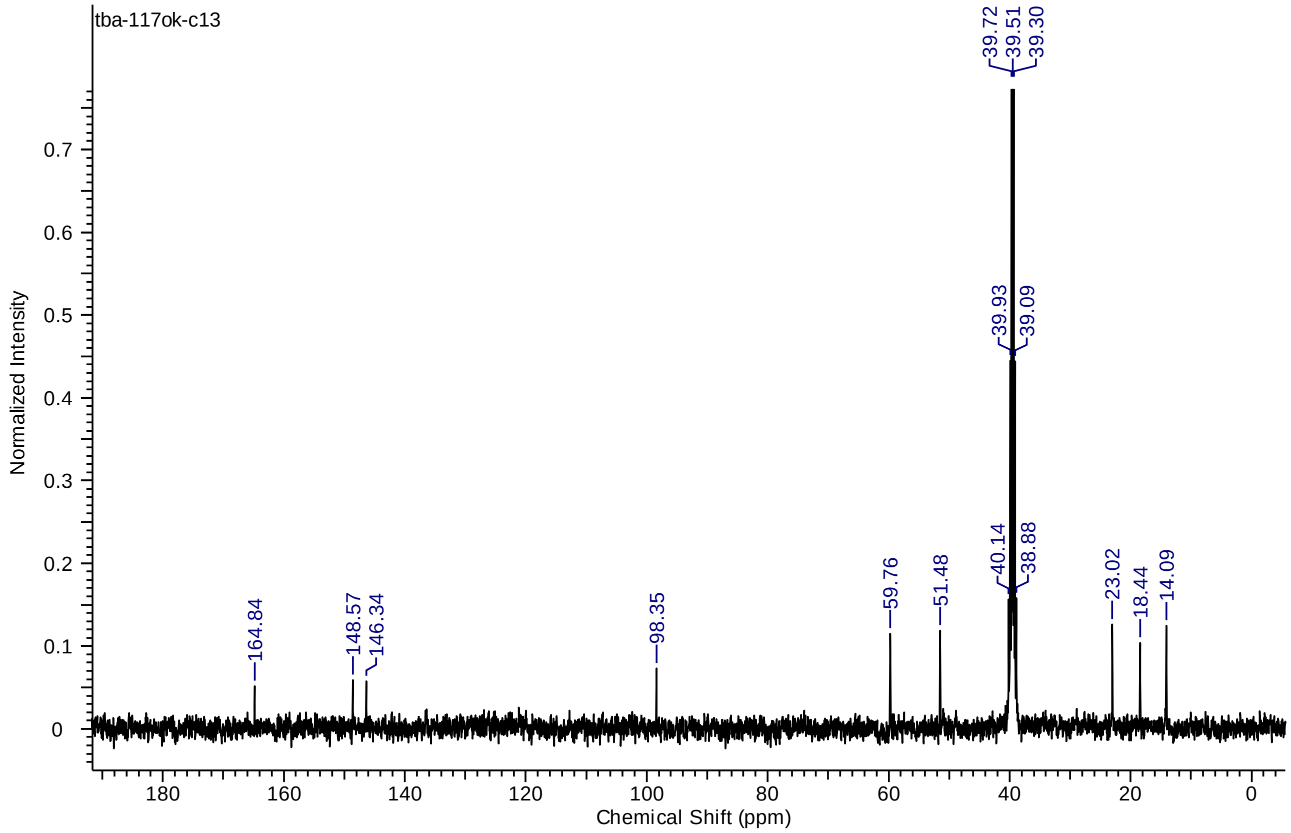
tba-117ok-c13
Normalized Intensity
39.72
39.51
39.30
39.93
39.09
40.14
38.88
164.84
148.57
146.34
98.35
59.76
51.48
23.02
18.44
14.09
Chemical Shift (ppm)

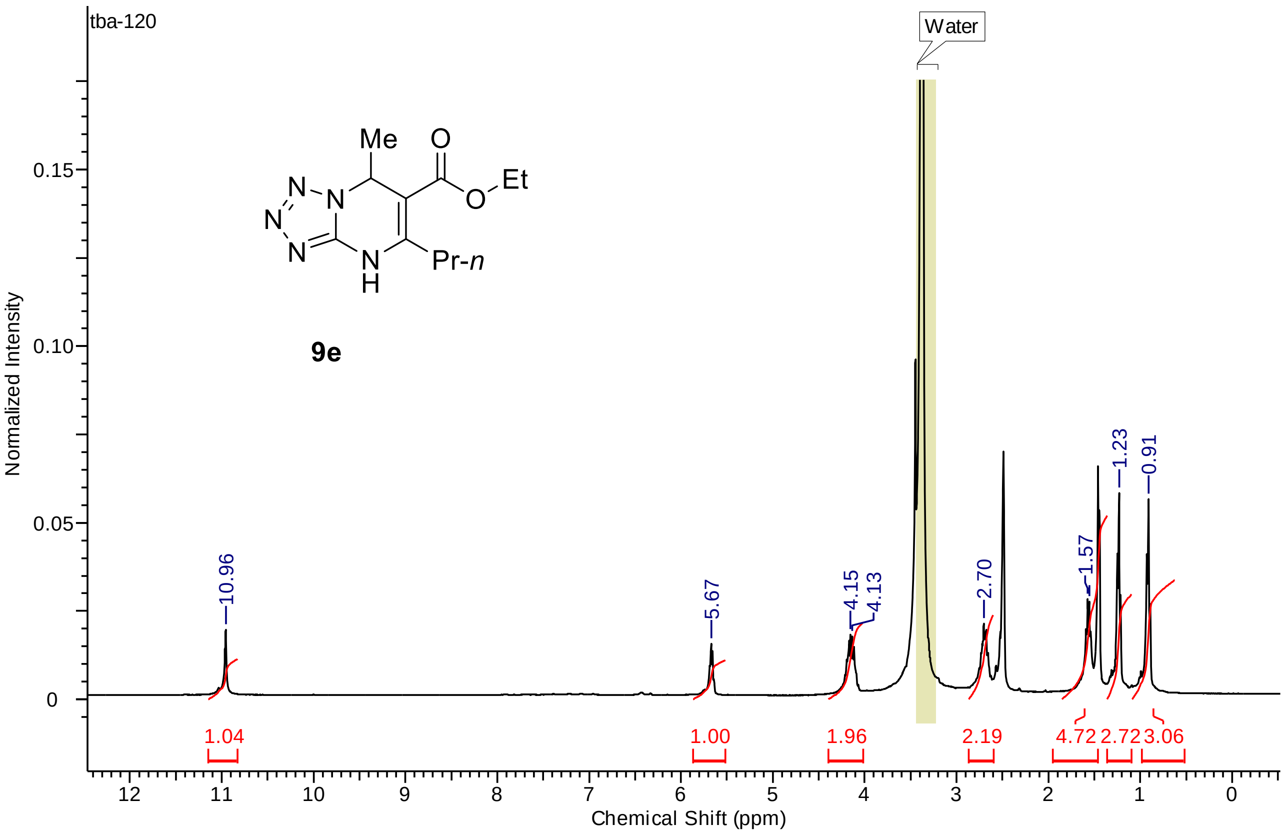

tba-120
Water
Me
O
N
N
N
N
O
Et
N
H
Pr-n
9e
10.96
5.67
4.15
4.13
2.70
1.57
1.23
0.91
1.04
1.00
1.96
2.19
4.72
2.72
3.06
Normalized Intensity
Chemical Shift (ppm)

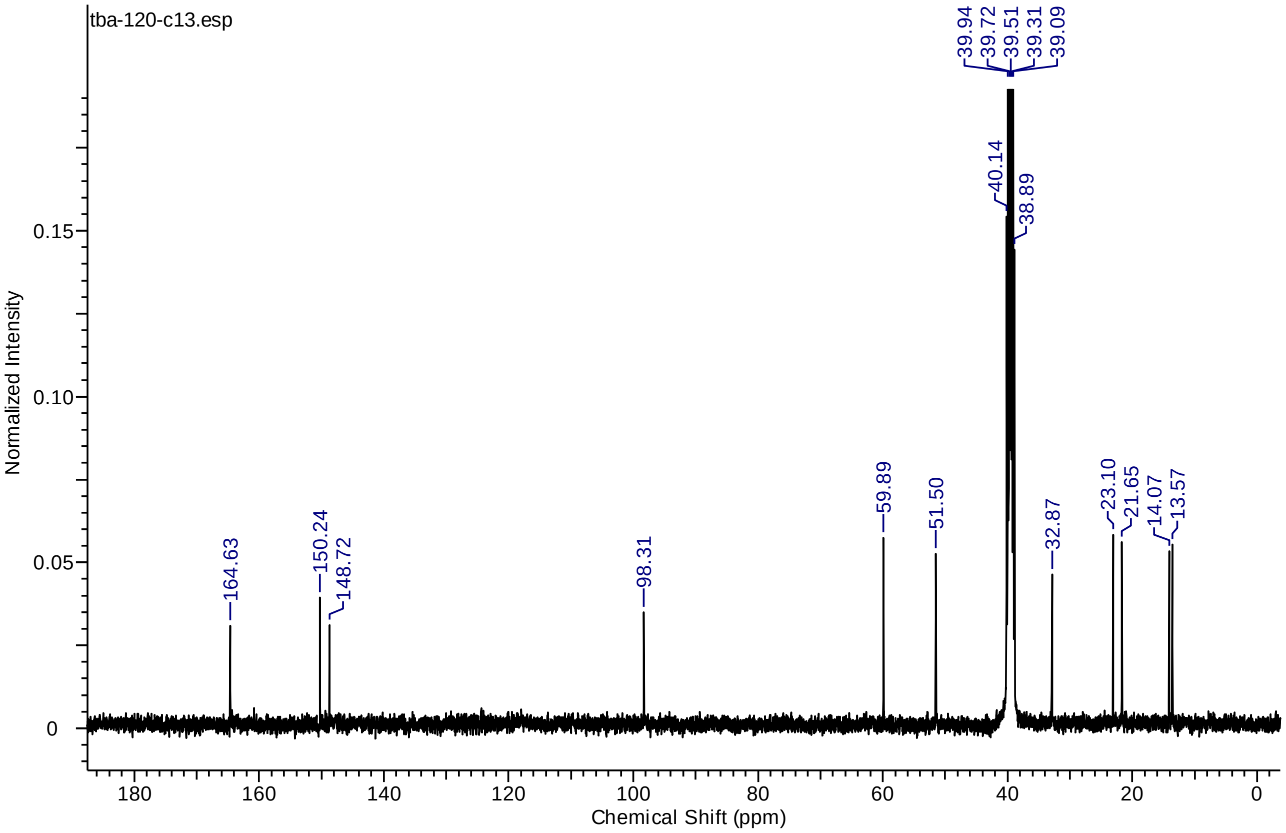

tba-120-c13.esp
39.94
39.72
39.51
39.31
39.09
40.14
38.89
164.63
150.24
148.72
98.31
59.89
51.50
32.87
23.10
21.65
14.07
13.57
Normalized Intensity
Chemical Shift (ppm)

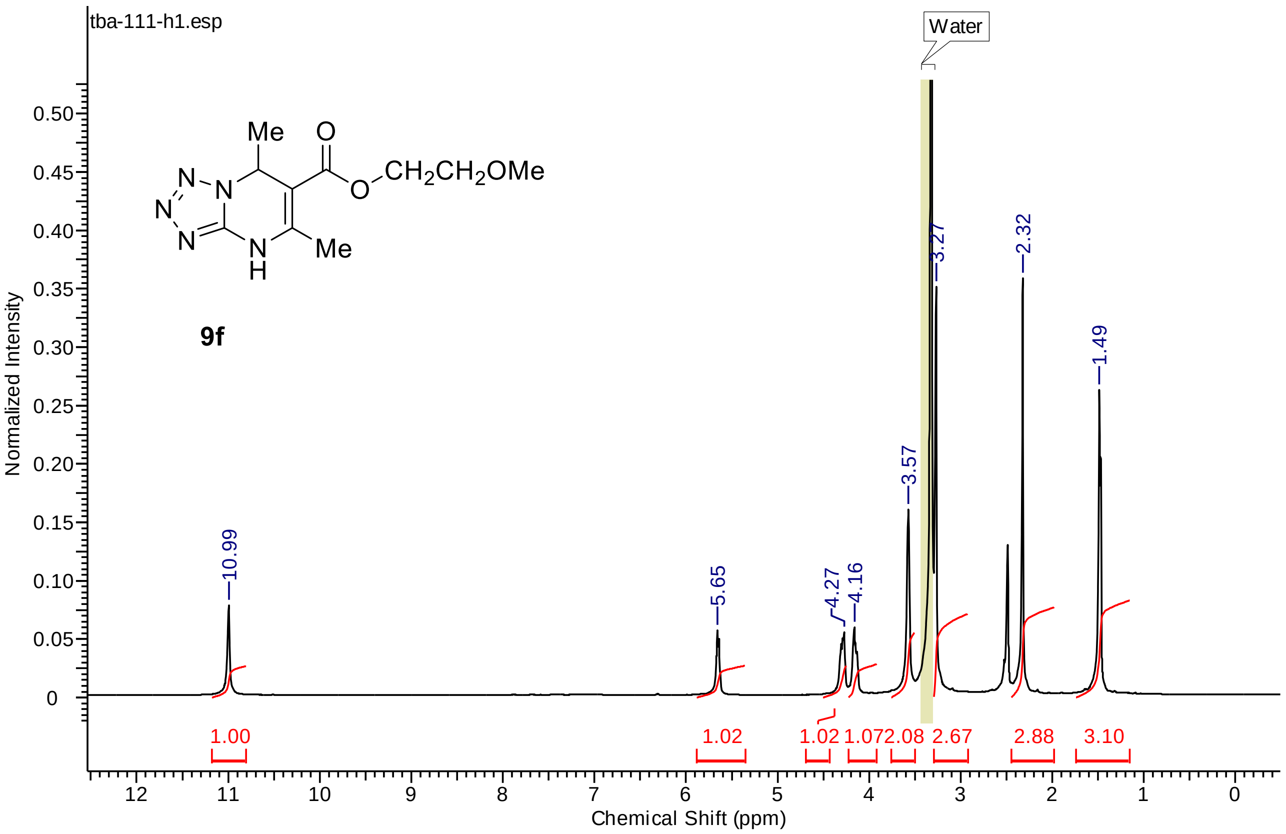
tba-111-h1.esp
Water
9f
Me
O
CH2CH2OMe
N
H
10.99
5.65
4.27
4.16
3.57
3.27
2.32
1.49
1.00
1.02
1.02
1.07
2.08
2.67
2.88
3.10
Normalized Intensity
Chemical Shift (ppm)

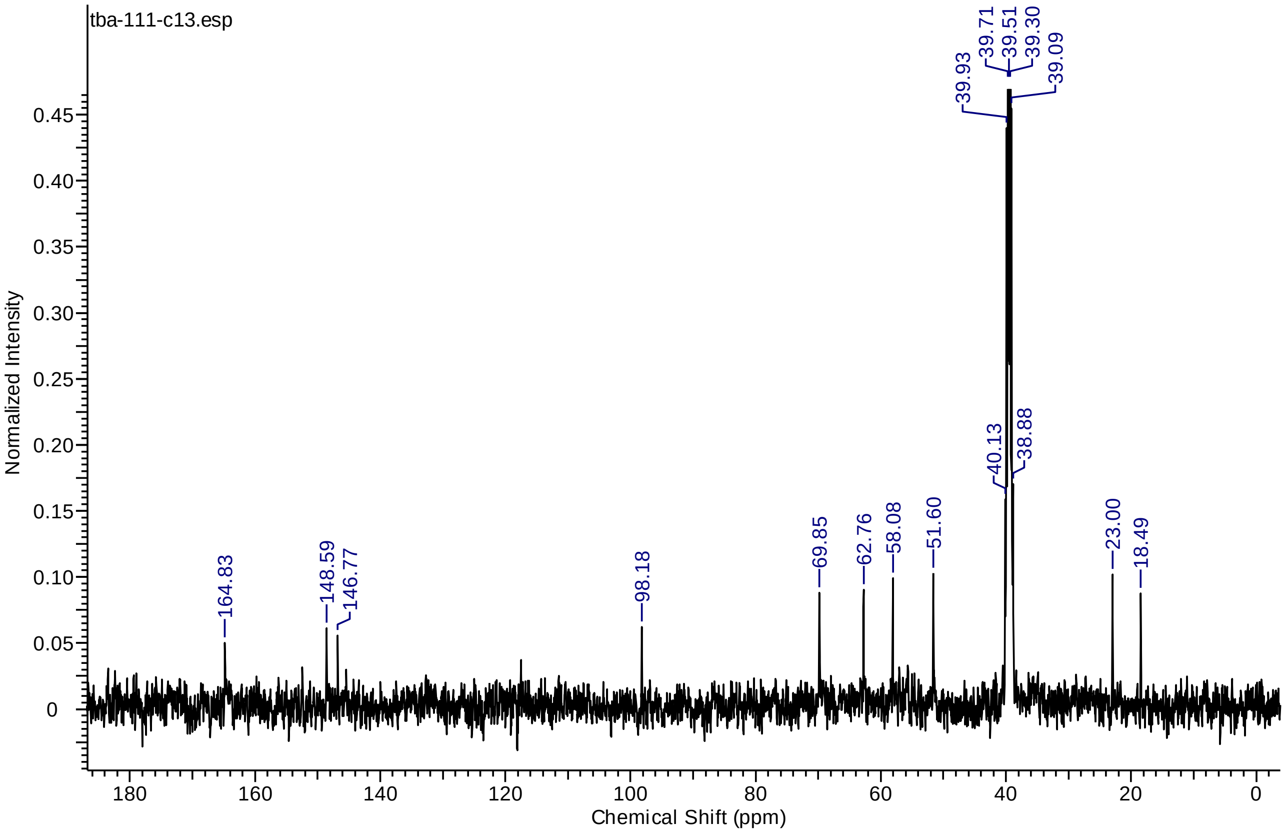
tba-111-c13.esp
164.83
148.59
146.77
98.18
69.85
62.76
58.08
51.60
40.13
39.93
39.71
39.51
39.30
39.09
38.88
23.00
18.49
Normalized Intensity
Chemical Shift (ppm)

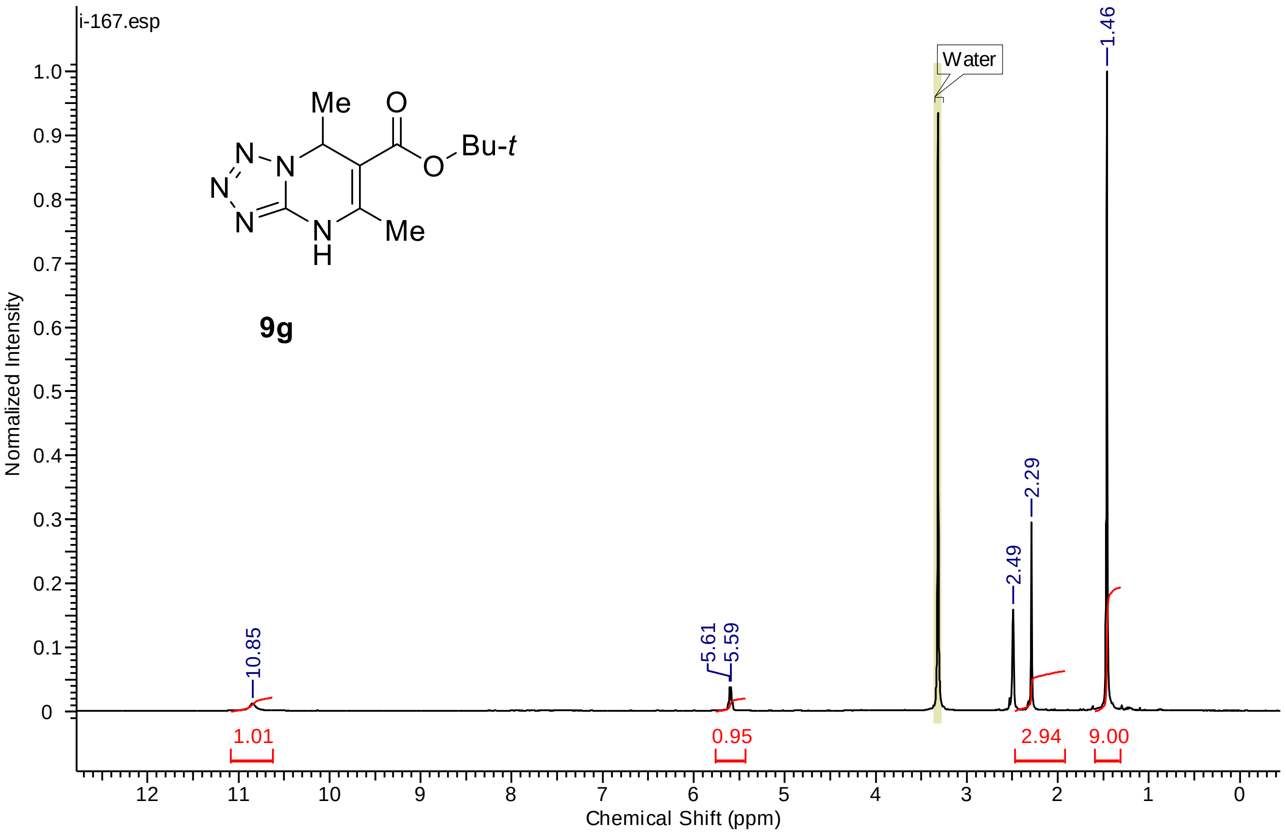
i-167.esp
Me
O
N
N
N
N
O
Bu-t
N
H
Me
9g
Water
10.85
5.61
5.59
2.49
2.29
1.46
1.01
0.95
2.94
9.00
Normalized Intensity
Chemical Shift (ppm)

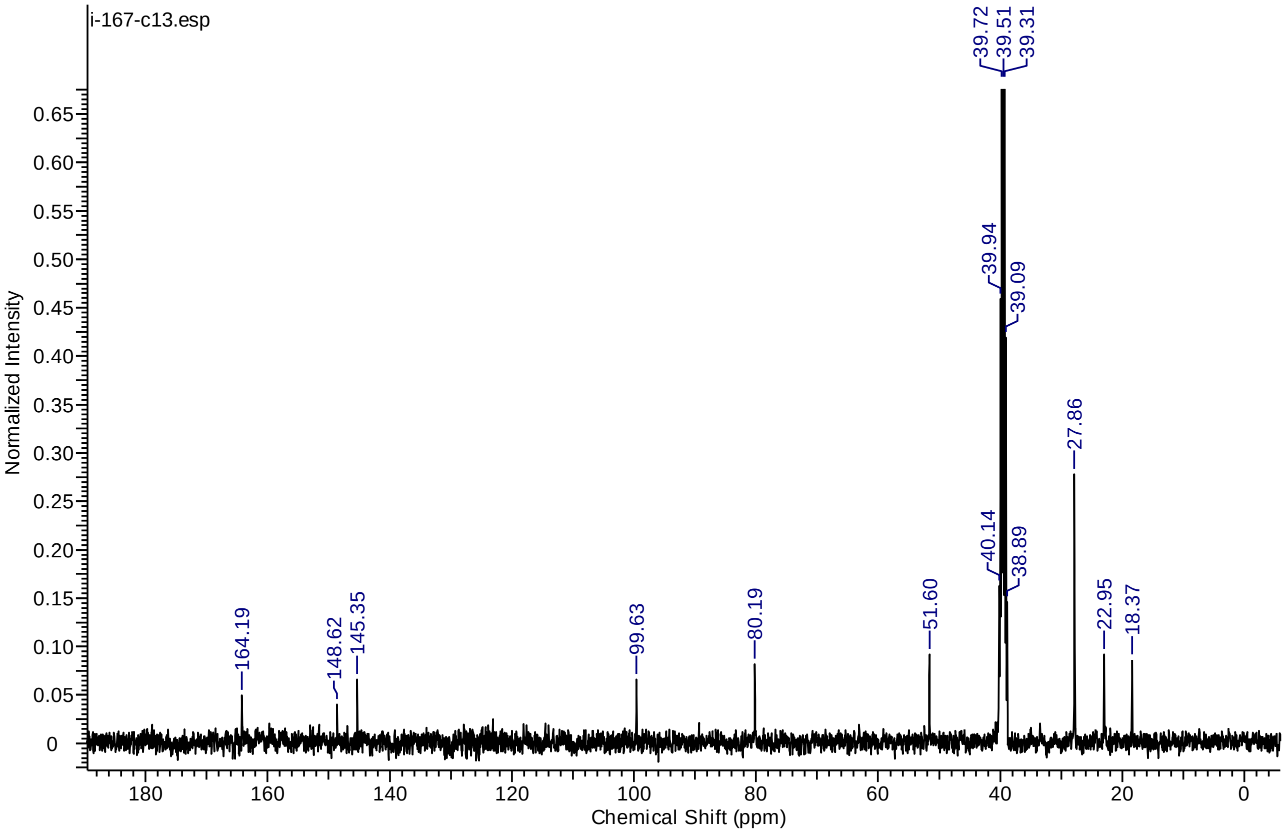
i-167-c13.esp
39.72
39.51
39.31
39.94
39.09
40.14
38.89
164.19
148.62
145.35
99.63
80.19
51.60
27.86
22.95
18.37
Normalized Intensity
Chemical Shift (ppm)

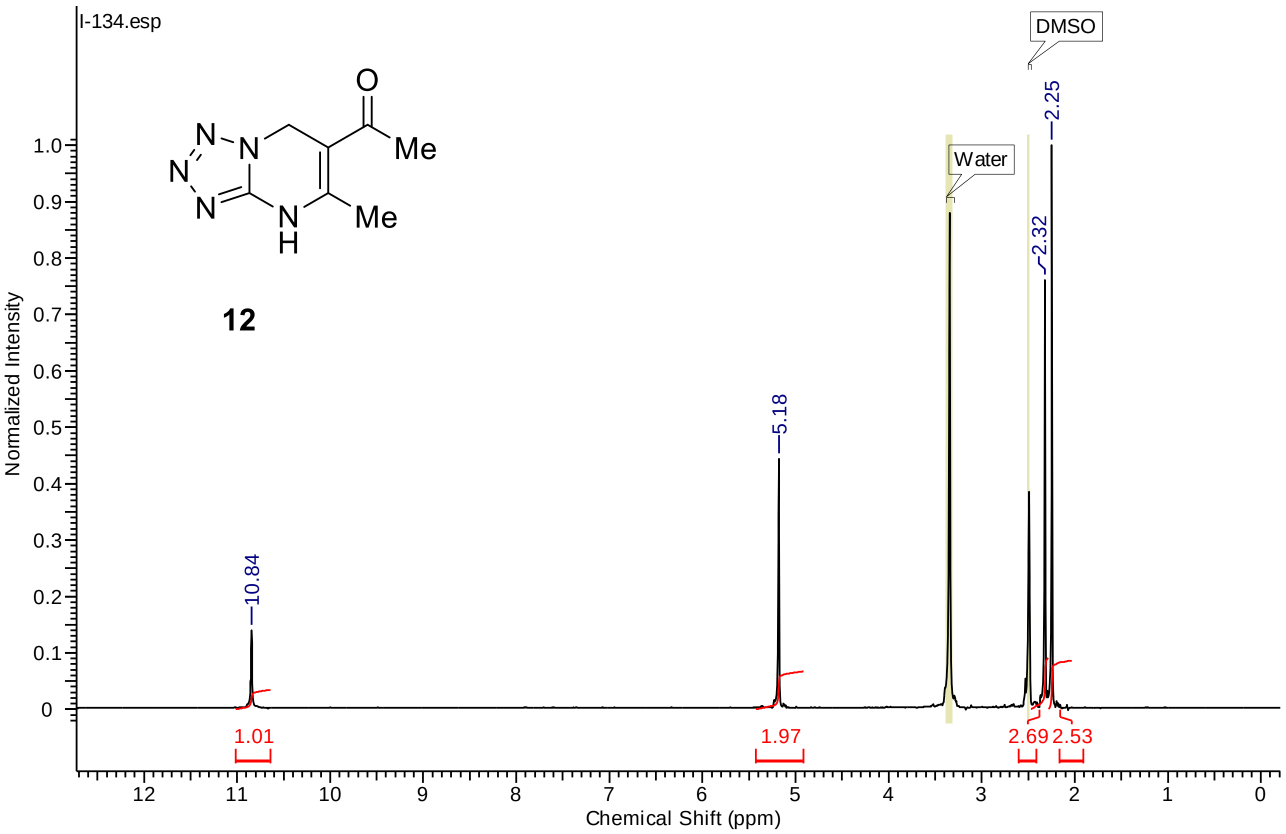

I-134.esp
12
Me
Me
DMSO
Water
10.84
5.18
2.32
2.25
1.01
1.97
2.69
2.53
Normalized Intensity
Chemical Shift (ppm)

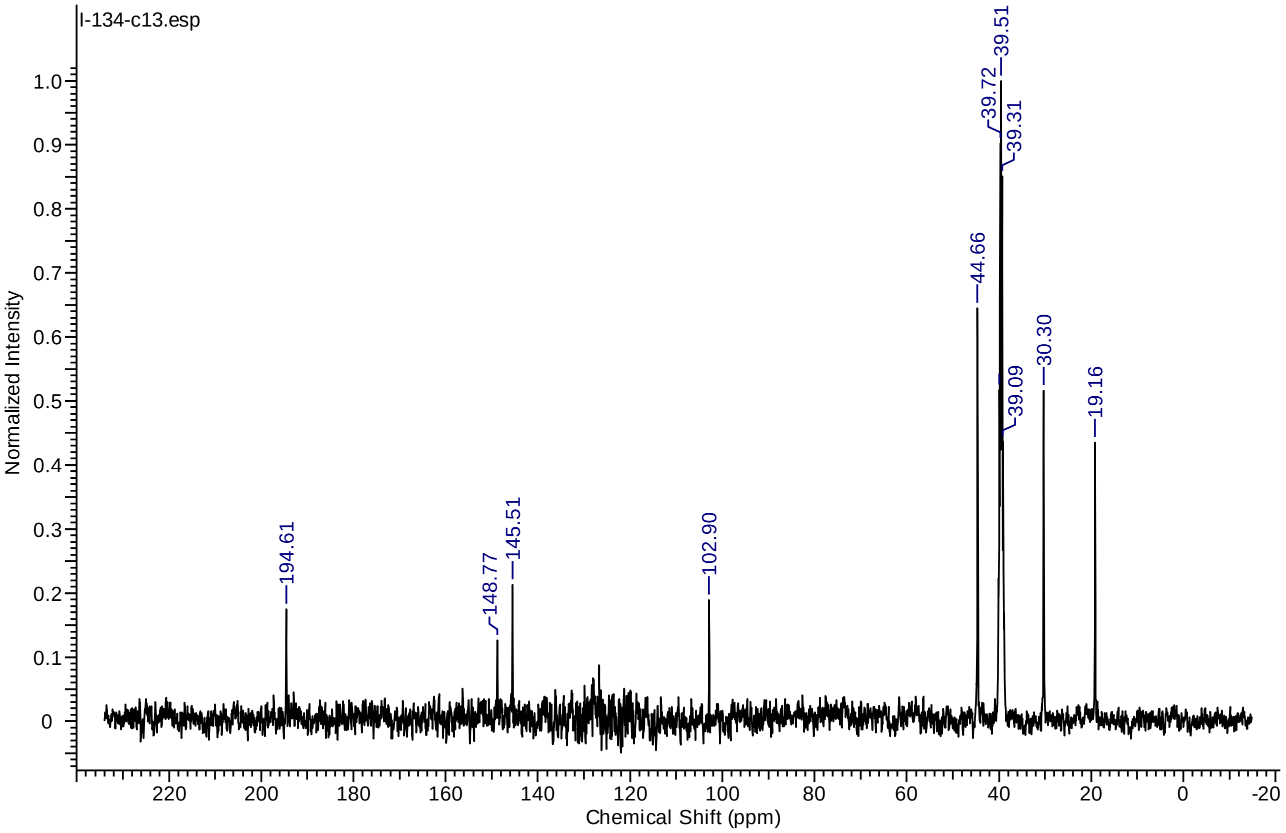

I-134-c13.esp
194.61
148.77
145.51
102.90
44.66
39.72
39.51
39.31
39.09
30.30
19.16
Normalized Intensity
Chemical Shift (ppm)

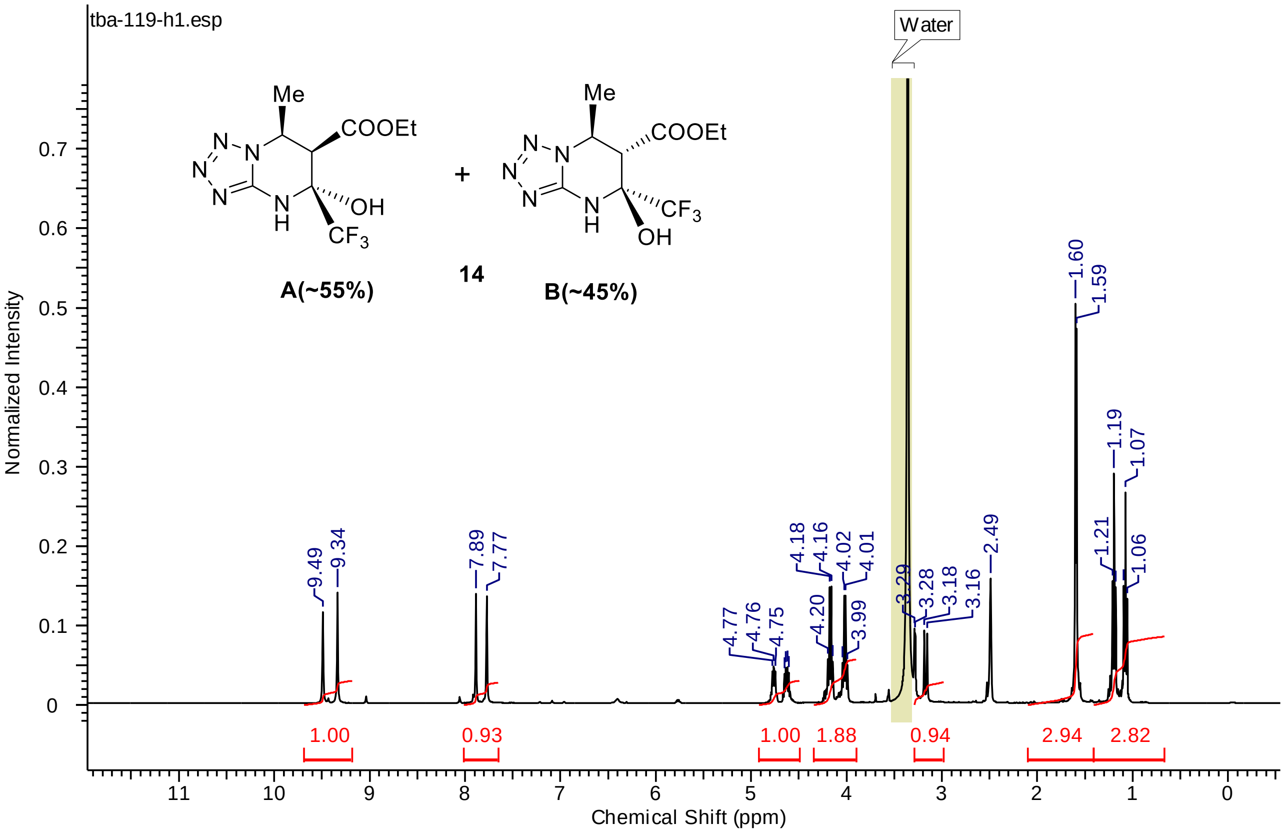
tba-119-h1.esp
Water
A(~55%)
14
B(~45%)
Normalized Intensity
Chemical Shift (ppm)

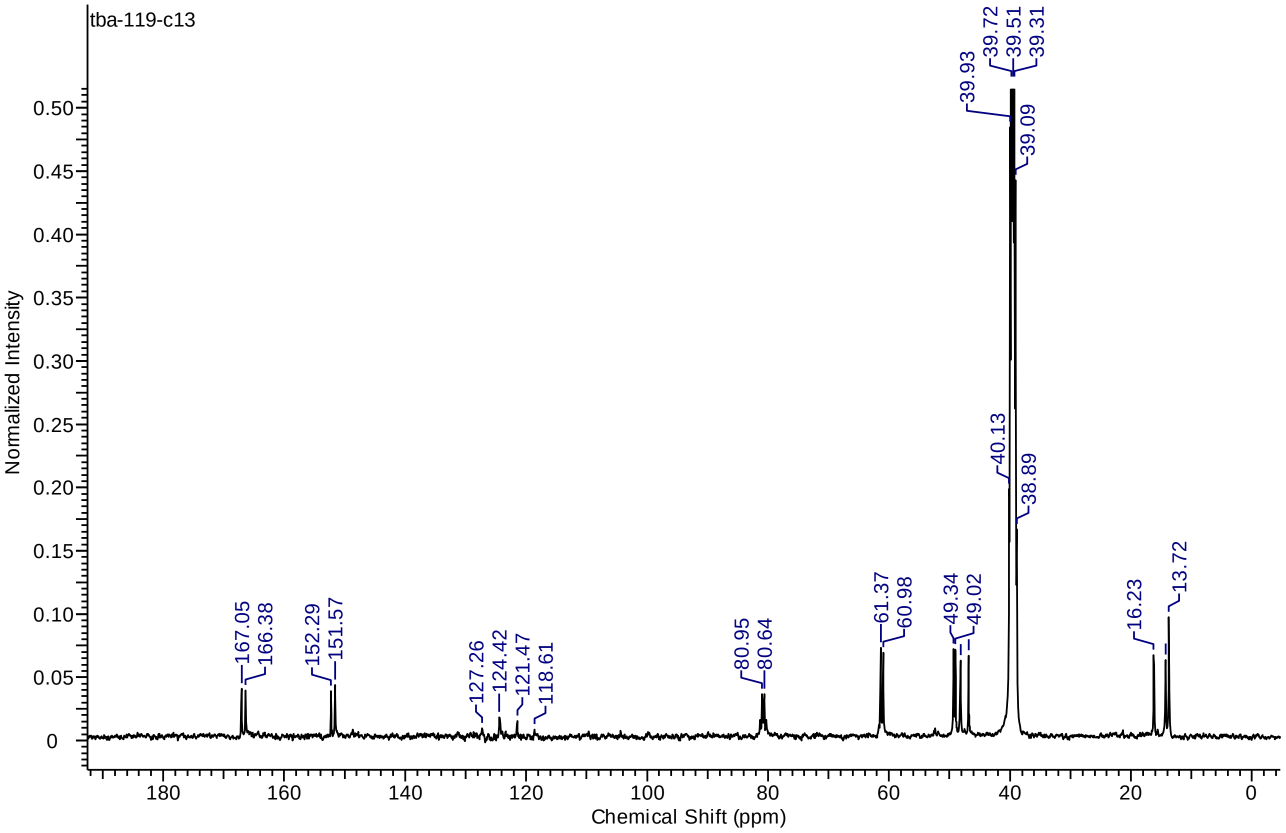
tba-119-c13
Normalized Intensity
Chemical Shift (ppm)

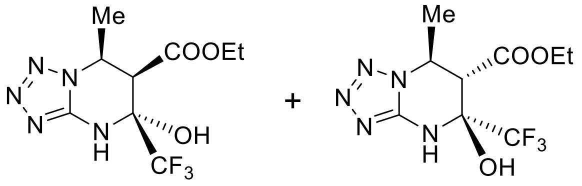

A(~55%) 14 B(~45%)

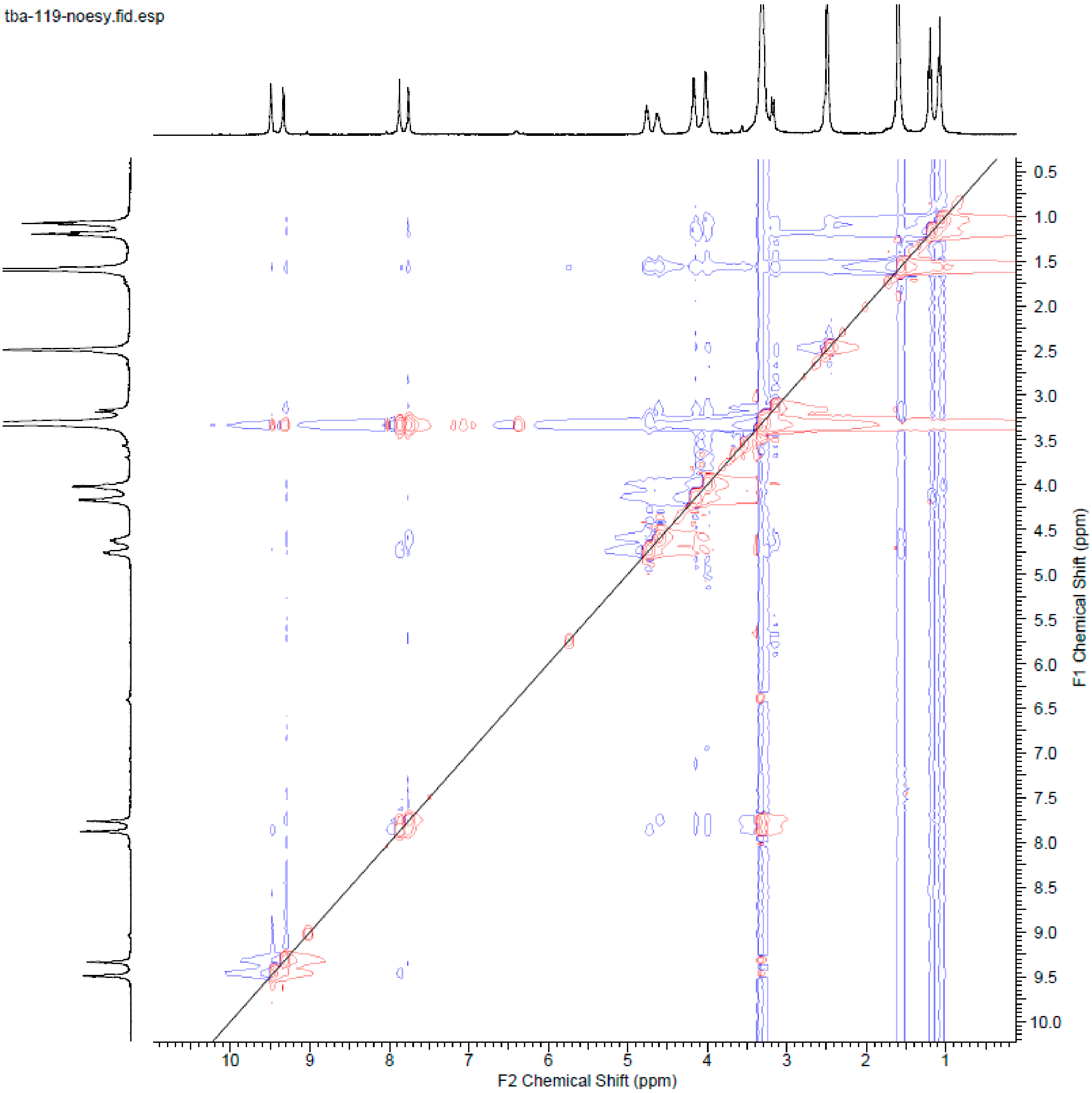
tba-119-noesy.fid.esp
F2 Chemical Shift (ppm)
F1 Chemical Shift (ppm)
